# TDAstats: R pipeline for computing persistent homology in topological data analysis

**DOI:** 10.21105/joss.00860

**Published:** 2018-08-08

**Authors:** Raoul R. Wadhwa, Drew F.K. Williamson, Andrew Dhawan, Jacob G. Scott

**Affiliations:** 1 Cleveland Clinic Lerner College of Medicine, Case Western Reserve University, Cleveland, OH 44195, USA; 2 Case Western Reserve University School of Medicine, Cleveland, OH 44106, USA; 3 Neurological Institute, Cleveland Clinic Foundation, Cleveland, OH 44195, USA; 4 Department of Translational Hematology and Oncology Research, Cleveland Clinic Foundation, Cleveland, OH 44195, USA

## Abstract

High-dimensional datasets are becoming more common in a variety of scientific fields. Well-known examples include next-generation sequencing in biology, patient health status in medicine, and computer vision in deep learning. Dimension reduction, using methods like principal component analysis (PCA), is a common preprocessing step for such datasets. However, while dimension reduction can save computing and human resources, it comes with the cost of significant information loss. Topological data analysis (TDA) aims to analyze the “shape” of high-dimensional datasets, without dimension reduction, by extracting features that are robust to small perturbations in data. Persistent features of a dataset can be used to describe it, and to compare it to other datasets. Visualization of persistent features can be done using topological barcodes or persistence diagrams (Figure 1). Application of TDA methods has granted greater insight into high-dimensional data (Lakshmikanth et al., 2017); one prominent example of this is its use to characterize a clinically relevant subgroup of breast cancer patients (Nicolau, Levine, & Carlsson, 2011). This is a particularly salient study as Nicolau et al. (2011) used a topological method, termed Progression Analysis of Disease, to identify a patient subgroup with 100% survival using that remains invisible to other clustering methods.

The TDAstats R package is a comprehensive pipeline for conducting TDA. Once data is loaded into R, TDAstats can calculate, visualize, and conduct nonparametric statistical inference on persistent homology. The Ripser C++ library (Bauer, 2015), benchmarked at approximately 40 times faster than comparable software, is wrapped using Rcpp (Eddelbuettel & Francois, 2011) for efficient computation of persistent homology. TDAstats generates topological barcodes and persistence diagrams using the ubiquitous ggplot2 library (Wickham, 2016), allowing use of ggplot2 functions to manipulate plots. This reduces the number of manual steps required to prepare publication-quality figures, thus enabling reproducible research (Sandve, Nekrutenko, Taylor, & Hovig, 2013). TDAstats also implements nonparametric hypothesis testing of persistent homology using a permutation test, first described by Robinson & Turner (2017). The permutation test uses statistical resampling to approximate the distribution of the test statistic assuming the null hypothesis is true. To our knowledge, TDAstats is the first library to implement this feature in the context of topological data analysis.

The primary barrier to using TDA is not mathematical comprehension. Although the algebraic topology that underlies TDA requires graduate-level study, the concepts necessary for application of TDA are far more intuitive. Rather, the barrier to entry is the lack of accessible, user-friendly software. TDAstats has an easy-to-use API with only 4 functions, each with only one or two intuitive parameters. Additionally, the provided vignettes cover its functionality with a comprehensive introduction and case study. Thus, even minimal knowledge of R will be sufficient to conduct TDA. We intend to use TDAstats to improve digit recognition algorithms, and hope that, with its efficient implementation and user-friendly API, a far larger set of students and researchers can now apply TDA to answer research questions.

## Figures and Tables

**Figure 1:**
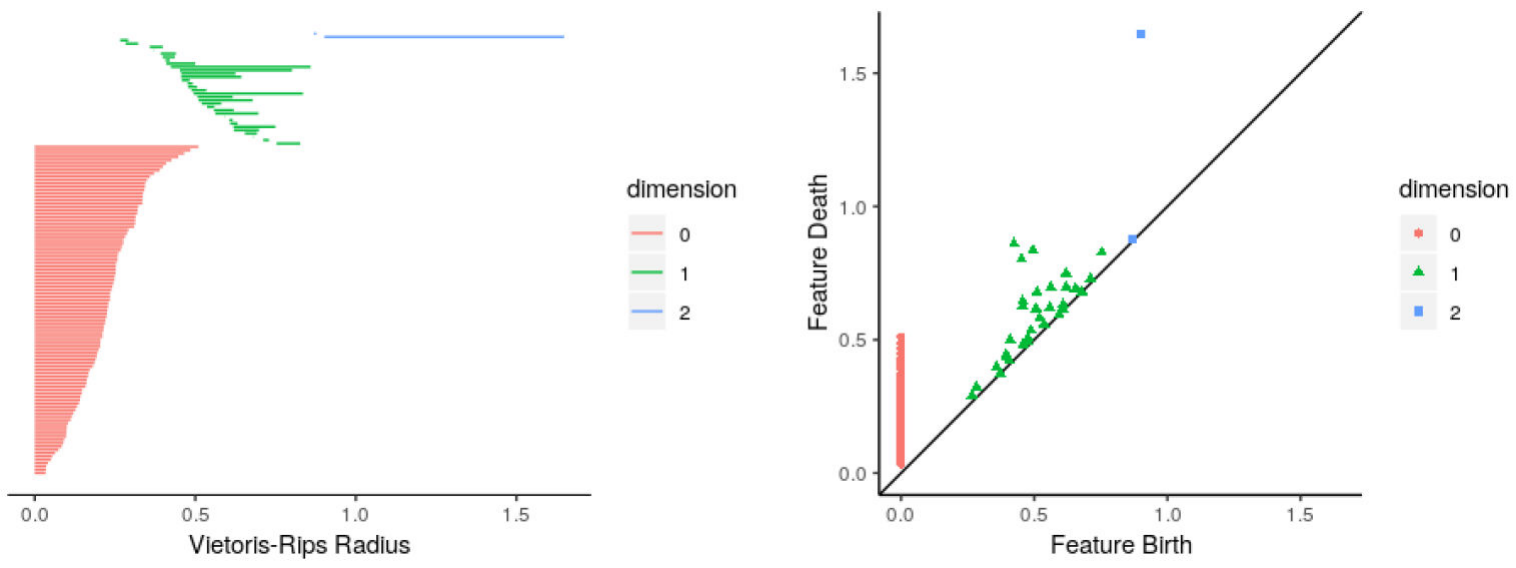
Topological barcode (left) and persistence diagram (right) of the sphere3d sample dataset included with TDAstats. The 0-cycles are colored red, the 1-cycles are colored green, and the 2-cycles are colored blue. For details on interpreting these plots, see Otter, Porter, Tillmann, Grindrod, & Harrington (2017).

## References

[R1] Bauer U (2015). Ripser: A lean c++ code for computation of vietoris-rips persistence barcodes. Retrieved from https://github.com/Ripser/ripser

[R2] Eddelbuettel D, & Francois R (2011). Rcpp: Seamless r and c++ integration. Journal of Statistical Software, 40(8), 1–18. doi:10.18637/jss.v040.i08

[R3] Lakshmikanth T, Olin A, Chen Y, Mikes J, Fredlund E, Remberger M, Omazic B, (2017). Mass cytometry and topological data analysis reveal immune parameters associated with complications after allogeneic stem cell transplantation. Cell Reports, 20, 2238–2250. doi:10.1016/j.celrep.2017.08.021 28854371

[R4] Nicolau M, Levine AJ, & Carlsson G (2011). Topology based data analysis identifies a subgroup of breast cancers with a unique mutational profile and excellent survival. Proceedings of the National Academy of Science, 108(17), 7265–7270. doi:10.1073/pnas.1102826108 PMC3084136 21482760

[R5] Otter N, Porter MA, Tillmann U, Grindrod P, & Harrington HA (2017). A roadmap for the computation of persistent homology. EPJ Data Science, 6, 17. doi:10.1140/epjds/s13688-017-0109-5 32025466 PMC6979512

[R6] Robinson A, & Turner K (2017). Hypothesis testing for topological data analysis. Journal of Applied and Computational Topology, 1(2), 241–261. doi:10.1007/s41468-017-0008-7

[R7] Sandve GK, Nekrutenko A, Taylor J, & Hovig E (2013). Ten simple rules for reproducible computational research. PLoS Computational Biology, 9(10), e1003285. doi: 10.1371/journal.pcbi.1003285 24204232 PMC3812051

[R8] Wickham H (2016). ggplot2: Elegant graphics for data analysis. Springer-Verlag New York Retrieved from https://ggplot2.tidyverse.org/

